# Thoroughbred Racehorses in Hong Kong Require Vitamin D Supplementation to Mitigate the Risk of Low Vitamin D Status

**DOI:** 10.3390/ani13132145

**Published:** 2023-06-29

**Authors:** Miranda C. M. Dosi, Chris M. Riggs, Jessica May, Adele Lee, Eugenio Cillan-Garcia, Joe Pagan, Bruce C. McGorum

**Affiliations:** 1 Royal (Dick) School of Veterinary Studies and The Roslin Institute, University of Edinburgh, Easter Bush Campus, Roslin EH25 9RG, UK; m.c.m.dosi@sms.ac.ed.uk (M.C.M.D.); info@eskvetconsultants.co.uk (E.C.-G.); 2 The Hong Kong Jockey Club Equine Welfare Research Foundation, Sha Tin Racecourse, New Territories, Hong Kong SAR, China; christopher.m.riggs@hkjc.org.hk (C.M.R.); jessicaseaburnemay@gmail.com (J.M.); adele.ct.lee@hotmail.com (A.L.); 3 Kentucky Equine Research, Versailles, KY 40383, USA; pagan@ker.com

**Keywords:** Thoroughbred, vitamin D, cholecalciferol, ergocalciferol, 25-hydroxyvitamin D_2_, 25-hydroxyvitamin D_3_

## Abstract

**Simple Summary:**

Vitamin D biology in equids is unique and poorly understood. Naturally managed (grazing) horses rely on dietary vitamin D_2_ (ergocalciferol) to provide adequate vitamin D, because endogenous ultraviolet radiation-mediated synthesis of vitamin D_3_ (cholecalciferol) is ineffective in this species. To test the hypothesis that the management of stabled, non-grazing racehorses is a risk factor for low vitamin D status, the vitamin D status of non-grazing Thoroughbred racehorses in Hong Kong (HK) and grazing Thoroughbred racehorses in the United Kingdom (UK) was compared. The HK horses had lower serum concentrations of 25-hydroxyvitamin D_2_ (25OHD_2_) and total 25-hydroxyvitamin D (total 25OHD: the index of vitamin D status), reflecting reduced dietary vitamin D_2_ intake. These data indicate that HK racehorses required dietary vitamin D_3_ supplementation to maintain adequate vitamin D status. The inverse relationship between the serum concentrations of 25OHD_2_ and 25OHD_3_, previously identified in humans, was observed for the first time in horses, indicating that further study is needed to determine the optimal form of dietary vitamin D supplementation for Thoroughbred racehorses.

**Abstract:**

There is a paucity of data relating to the vitamin D status of racehorses. We hypothesised that the management of racehorses in Hong Kong (HK) predisposes to low vitamin D status unless they receive dietary supplementation. Serum concentrations of 25-hydroxyvitamin D_2_ (25OHD_2_), 25-hydroxyvitamin D_3_ (25OHD_3_) and total 25-hydroxyvitamin D (total 25OHD) for 79 non-grazing HK racehorses were compared with those for 22 racehorses training in the United Kingdom (UK) that grazed for ≥1 h/d, and for which published data exists. A nested group of 41 HK horses was sampled twice to determine the effect of the duration in HK on vitamin D status. The HK horses had significantly lower serum concentrations of total 25OHD and 25OHD_2_ than the UK horses; 25OHD_2_ was undetectable in 15/79 HK sera and serum concentrations of 25OHD_2_ declined with the duration in HK. The main determinants of vitamin D status were assessed using linear regression; the retained variables were the 25OHD_3_ concentration and the duration in HK. The inverse relationship between the serum concentrations of 25OHD_2_ and 25OHD_3_, previously identified in humans, was observed for the first time in horses. In conclusion, HK racehorses have low serum 25OHD_2_ and total 25OHD concentrations and rely on D_3_ supplementation to maintain adequate vitamin D status. Further study is required to determine the optimal form of dietary vitamin D supplementation for Thoroughbred racehorses.

## 1. Introduction

Vitamin D has important roles in calcium homeostasis, bone health and modulation of immune and inflammatory responses through endocrine and paracrine effects in many body tissues [1,2,3]. Vitamin D exists in two forms, namely vitamin D_2_ (D_2_: ergocalciferol), which is produced by fungi growing on plants [4,5], and vitamin D_3_ (D_3_: cholecalciferol) which, in some species, can be synthesised within the skin through the action of ultraviolet B (UVB) radiation on 7-dehydrocholesterol [6]. D_2_ and D_3_ may also be derived from dietary supplementation. To become physiologically active, these compounds must undergo a first hydroxylation in the liver. The serum concentrations of the resultant 25-hydroxyvitamin D_2_ (25OHD_2_) and 25-hydroxyvitamin D_3_ (25OHD_3_), and the sum of 25OHD_2_ and 25OHD_3_ (total 25OHD), are used as indicators of the vitamin D status in humans and horses [7,8]. The 25OHD compounds undergo a final hydroxylation in the kidneys to produce the biologically active moieties 1,25-dihydroxyvitamin D_2_ and 1,25-dihydroxyvitamin D_3_.

Horses appear to have a unique vitamin D biology. In the absence of vitamin D supplementation, they rely on 25OHD_2_ derived from UVB irradiated dietary forage because they synthesise no or negligible quantities of D_3_ in their skin through the action of solar UVB radiation on 7-dehydrocholesterol [8,9,10]. Consistent with this, D_3_ could not be detected in equine skin following experimental UVB exposure [11]. Furthermore, serum 25OHD_3_ concentrations remained low or undetectable throughout the year in healthy non-supplemented grazing horses in New Zealand, the United Kingdom (UK) and Thailand (respectively, 40° S, 56° N and 15° N) [8,9,10].

Hymøller and Jensen [1] concluded their article entitled ‘*We know next to nothing about vitamin D in horses*’ by emphasising the need to assess vitamin D requirements of horses under different management conditions and for horses of different breeds, ages and genders. In particular, there is a paucity of data relating to vitamin D status in racehorses. The present study assessed the vitamin D status of racehorses in training in Hong Kong (HK) to investigate the hypothesis that the horses’ management and athletic activity predispose them to having low vitamin D status unless they receive appropriate dietary vitamin D supplementation. HK horses are stabled with limited UVB exposure, have no access to grazing and are fed restricted amounts of forage and commercial feed containing various amounts of supplemental D_3_. HK horses undergo strenuous athletic activity, which has been reported to reduce vitamin D metabolites in healthy horses [10,12]. Additionally, reductions in serum 25OHD concentrations have been reported in horses with inflammatory diseases, including mild-moderate equine asthma, which is prevalent in racehorses [13,14].

A cross-sectional study was used to determine the vitamin D status of non-grazing HK racehorses (*n* = 79). Data were compared with those for racehorses training in the UK that grazed for ≥1 h/d, and with those reported in previous equine studies. We hypothesised that the UK horses would have higher serum 25OHD_2_ concentrations than the HK horses given that grazing provides a natural source of dietary D_2_ [4,8]. Additionally, a nested group of HK horses was sampled twice to determine the effect of the duration in HK on vitamin D status. The effects of country of origin, duration in HK, premises, age and gender on the vitamin D status of HK horses were also assessed.

## 2. Materials and Methods

### 2.1. Horses

The 79 HK Thoroughbred horses comprised 5 colts, 69 geldings and 5 females. At the time of the first (or unique) sampling, mean age was 4.3 years (range 2.4–7.9). Moreover, to investigate the effect of duration in HK on vitamin D status, a nested group of 41 HK horses was sampled at two time points, a median of 62 days (range 20–152) apart. HK horses were imported from New Zealand (*n* = 28), Australia (*n* = 28), the UK (*n* = 12), Ireland (*n* = 5), France (*n* = 4) and USA (*n* = 2). Duration in HK before the first or unique sampling ranged from 2 weeks to 4.7 years (median 0.8 years, Q1–Q3 14 days–1.7 years). HK horses were stabled with 19 different trainers in a single complex in an urban environment. Trainers all shared the same training resources although they employed different management practices and fed the horses different diets. Horses effectively had no access to grazing and typically ≤ 30 min sunlight exposure while training and during a similar time of in-hand walking daily. They were fed approximately 2–2.5% bw as dry matter (DM), including restricted amounts (likely averaging < 1% bw as DM) of forage, which was typically timothy (*Phleum pratense*) hay imported from North America, with some receiving chaff and/or haylage imported from New Zealand. They also received 6–7 kg/d of commercial feeds supplemented with 1100–2100 IU/kg (as fed) of D_3_, which provides in excess of the current minimum vitamin D requirement of 6.6 IU/kg BW/d [15]. Food was stored in air-conditioned stores, which likely retarded fungal growth on forage, possibly reducing the forage D_2_ content. Some horses also received 1 kg/d freshly cut grass. The UK horses (*n* = 22) had a mean age of 7 years (range 3–11) and comprised 2 females and 20 geldings. They were stabled on a single premises in Scotland, were in training and were fed 8–10 kg/d haylage and a commercial concentrate feed providing 6600–8800 IU/d D_3,_ which exceeds the current minimum requirement of 6.6 IU/kg/d [15]. They grazed on pasture for ≥1 h/d, wearing a rug.

### 2.2. Serum Samples

Venous blood was collected from all horses and transferred into plain vacutainers. Blood samples were residues of samples taken for clinical purposes as part of the horses’ routine veterinary management with trainer consent and ethical approval from the HKJC Ethical Review Committee and the Edinburgh School of Veterinary Medicine Ethical Review Committee. Serum was harvested and frozen at −20 °C or −80 °C prior to analysis. Serum from HK horses was shipped on dry ice to the UK for analysis.

### 2.3. Vitamin D Analyses

Serum concentrations of 25OHD_2_, 25OHD_3_ and total 25OHD were quantified by a commercial laboratory (Biolab Medical Unit, London, UK) using high-performance liquid chromatography with UV detection using reagents supplied by Chromsystems Instruments and Chemicals GMBH (Heimburgstrasse 3, 81243 Munchen, Germany). The lower limit of detection for 25OHD_2_ and 25OHD_3_ was 0.5 nmol/L. For statistical analysis, values for 25OHD_2_ that were below this detection limit were recorded as 0.49 nmol/L.

### 2.4. Statistical Analysis

Most data were non-normally distributed. Intergroup paired comparisons (e.g., 22 UK horses versus 79 HK horses) were made using the Mann–Whitney test. Multiple-group comparisons (e.g., country of origin) were made using the Kruskal–Wallis test. Intragroup comparisons (e.g., first versus second sampling) were made using the Wilcoxon rank test. For the regression analysis, non-normally distributed data were ln transformed, although 25OHD_2_ remained non-normally distributed after this transformation. Multivariate linear regression was used to assess the effect of duration in HK, country of origin, stable, age and gender on vitamin D status. In the univariate model, only variables that had *p* < 0.2 were put forward into the final model. Statistical analyses were conducted with SPSS 26 (IBM).

## 3. Results

### 3.1. Intergroup Comparisons

**Figure 1:**
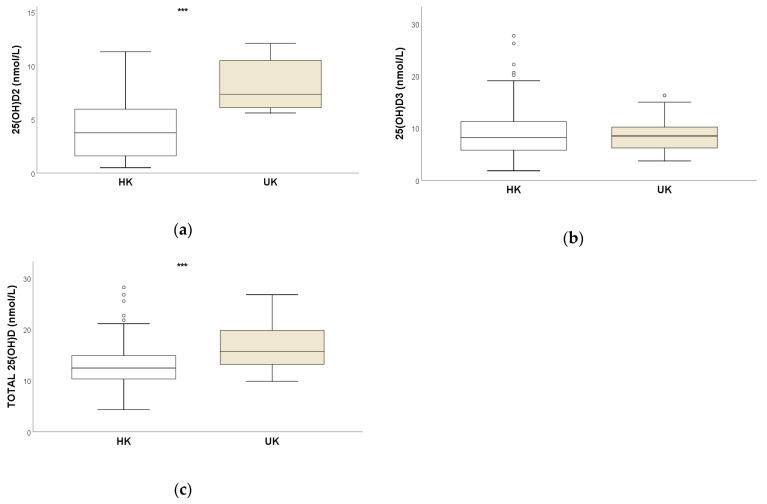
Box and whisker plots comparing serum concentrations (nmol/L) of (**a**) 25OHD_2_, (**b**) 25OHD_3_ and (**c**) total 25OHD for HK (*n* = 79) and UK (*n* = 22) horses. *** Significant inter-group difference (*p* < 0.001).

There was no significant inter-group age difference. Descriptive statistics for serum concentrations of 25OHD_2_, 25OHD_3_ and total 25OHD for the UK and the HK horses are presented in Table 1 and Figure 1. The UK horses had significantly higher concentrations of 25OHD_2_ and total 25OHD (both *p* < 0.001; Mann–Whitney) (Table 1 and Figure 1). The 25OHD_2_ was detectable in all the UK horse sera but was below the limit of detection for 15/79 (19%) of the HK horse sera.

### 3.2. Intragroup Comparisons

**Figure 2:**
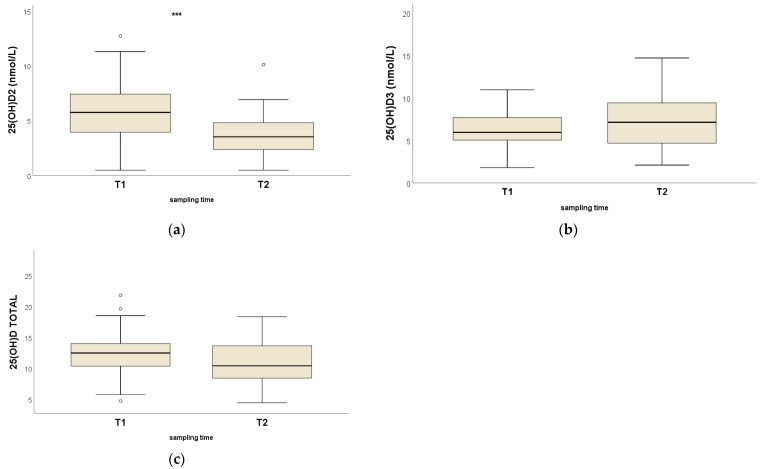
Box and whisker plots for serum concentrations (nmol/L) of (**a**) 25OHD_2_, (**b**) 25OHD_3_ and (**c**) total 25OHD for HK racehorses (*n* = 41) at two different time points. *** Significant intragroup difference (*p* < 0.001).

Serum concentrations of 25OHD_2_ for the HK horses decreased significantly between the first and second sampling points (*p* < 0.001; Wilcoxon rank test), while 25OHD_3_ (*p* = 0.12) and total 25OHD (*p* = 0.35) concentrations were not significantly different (Table 2 and Figure 2).

### 3.3. Univariate and Multivariate Linear Regression

**Figure 3:**
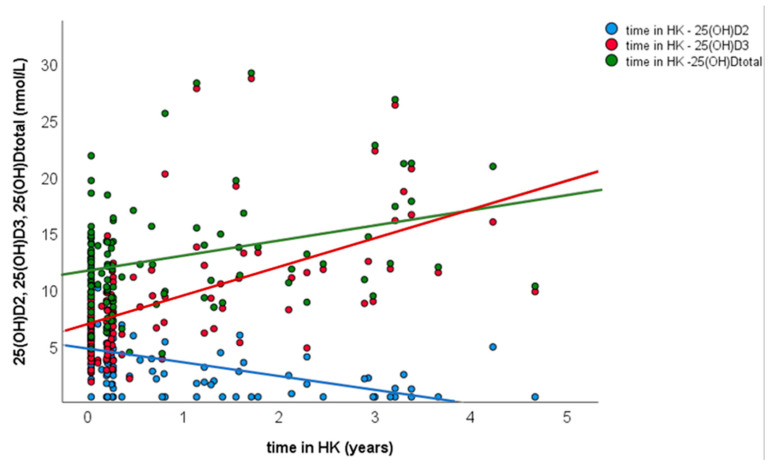
Scatter plot representing the association between duration in HK (years) and serum concentrations (nmol/L) of 25OHD_2_ (blue), 25OHD_3_ (red) and total 25OHD (green) for HK horses (*n* = 79).

For univariate linear regression, 25OHD concentrations were transformed to a logarithmic scale (ln) and ln total 25OHD concentration of the first samples for the 79 HK horses used as outcome, and, as predictors, duration in HK, ln25OHD_2_ concentration, ln25OHD_3_ concentration, trainer and country of origin. Only variables with *p* < 0.2 in the univariate analysis, namely duration in HK and ln25OHD_3_ concentration, were put forward into the final multivariate analysis (Table 3). Ln25OHD_3_ and duration in HK were retained in the final model (*p* < 0.01), with 72% of the variability in total 25OHD concentrations being explained by the 25OHD_3_ concentration and the duration in HK (R^2^ = 0.722) (Table 3). There was a positive relationship between the duration in HK and the 25OHD_3_ concentration (*p* < 0.01, β = 0.985), and a negative relationship between the duration in HK and the 25OHD_2_ concentration (*p* < 0.01, β = −0.330) (Figure 3).

### 3.4. Correlation of 25OHD_2_, 25OHD_3_ and Total 25OHD

**Figure 4:**
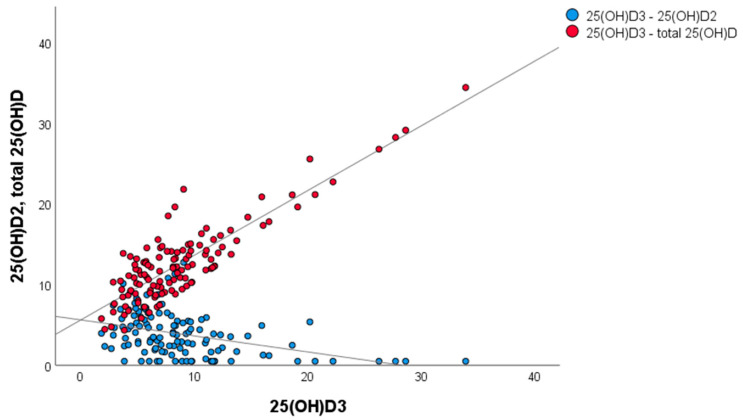
Scatter plot for serum concentrations (nmol/L) of 25OHD_3_ (x axis) and 25OHD_2_ and total 25OHD (both on y axis) for HK horses (*n* = 120 samples), highlighting the negative relationship between 25OHD_3_ and 25OHD_2_ concentrations (blue), and positive relationship between 25OHD_3_ and total 25OHD concentrations (red).

When data for all 120 HK samples were considered (Figure 4), there was a positive relationship between total 25OHD and 25OHD_3_ concentrations (β = 0.800, *p* < 0.01), and a negative relationship between 25OHD_3_ and 25OHD_2_ concentrations (β = −0.453, *p* < 0.01).

## 4. Discussion

### 4.1. Vitamin D Status of UK and HK Thoroughbred Racehorses

This study contributes to our knowledge regarding the vitamin D status of Thoroughbred racehorses in training in HK and in the UK. Serum concentrations of total 25OHD for HK (median 12.5, range 4.3–29.1 nmol/L) and UK (15.8, range 9.9–26.8 nmol/L) horses were largely consistent with previous reports that healthy horses from a wide range of breeds, ages, management systems and latitudes, and in different seasons, have total 25OHD concentrations < 25 nmol/L [1,8,9,16,17,18]. These values are considerably lower than those for other species, reflecting the peculiarities in equid vitamin D biology [16,19,20,21,22]. Consistent with published equine data [summarised by 2], a wide range of total 25OHD concentrations was observed in the HK and UK horses, despite each group having fairly similar management practices. Some horses, predominantly in the HK group, had very low total 25OHD concentrations, with the lowest being 4.3 nmol/L. While the threshold serum concentration of total 25OHD which reflects adequacy of vitamin D for maintenance of optimal health and athletic performance is currently unknown, it is possible that those horses with the lowest total 25OHD concentrations may have had inadequate vitamin D status and may have benefitted from additional vitamin D supplementation. The main limitation of this study was the inability, for practical and logistical reasons, to quantify accurately dietary D_2_ (forage and grazing) and D_3_ (supplementation) intakes for all of the individual horses in the study.

HK horses had significantly lower serum concentrations of total 25OHD than UK horses. This was attributable largely to their very low serum concentrations of 25OHD_2_; indeed, 25OHD_2_ was undetectable (<0.5 nmol/L) in 15/79 (19%) of the HK horse sera. Furthermore, there was a significant temporal reduction in 25OHD_2_ concentrations in the nested group of HK horses that were sampled on two occasions and a negative statistical association between the 25OHD_2_ concentrations and the duration in HK. As 25OHD_2_ is derived from D_2,_ which is produced by fungi growing on UVB irradiated forages [5], the low 25OHD_2_ concentrations in HK horses likely reflect the absence of grazing and restricted intake of sun-cured forage. Consistent with this hypothesis, serum concentrations of 25OHD_2_ for HK horses were lower than previously reported for grazing and forage fed horses [8,9,17]. The low 25OHD_2_ and total 25OHD concentrations in the HK horses may also reflect the effects of intense athletic activity, and/or lower airway inflammation, which is prevalent in Thoroughbred racehorses [10,12,13,14].

This study highlights the importance of dietary D_3_ supplementation in compensating for inadequate D_2_ intake and maintaining adequate vitamin D status in HK racehorses. Considering the fact that HK horses have very low or undetected 25OHD_2_ concentrations, it is logical that in the multivariate analysis of factors affecting vitamin D status (total 25OHD), the only variables retained in the final model were the 25OHD_3_ concentration and the duration in HK.

### 4.2. In Racehorses, Serum 25OHD_3_ Is Probably Derived Solely from Dietary D_3_ Supplementation

It is highly probable that the 25OHD_3_ detected in the HK and UK horse sera was derived solely from dietary D_3_ supplementation, as reported in other equine studies [8,9,17]. Endogenous UVB-mediated 25OHD_3_ synthesis can be ruled out because a recent ex vivo study demonstrated that, unlike ovine skin biopsies, equine skin biopsies produce negligible quantities of vitamin D_3_ when exposed to the wavelengths of UVB light that induce non-enzymatic conversion of 7-dehydrocholesterol to vitamin D_3_ in other species [11]. Consequently, serum 25OHD_3_ concentrations remained low or undetectable throughout the year in healthy non-supplemented grazing horses in New Zealand, the United Kingdom (UK) and Thailand (respectively, 40° S, 56° N and 15° N) [8,9,10]. The horse’s inability to endogenously synthesise vitamin D3 contrasts with other domestic herbivores (sheep, cattle and goats) that exhibit effective endogenous vitamin D3 synthesis [23,24,25]. Furthermore, the horses in this study had only limited UVB exposure at the time of sampling. Most (89%) of the HK samples were collected during autumn–winter (November to February), when the local UV index (https://www.hko.gov.hk/en/wxinfo/uvinfo/uvinfo.html data gathered by HK observatory website accessed on the 2 April 2023) was below that required for endogenous 25OHD_3_ synthesis in other species [26]. Similarly, UVB radiation is insufficient for endogenous 25OHD_3_ synthesis in UK horses given the latitude (56° N) and the month (March) of sampling for the UK horses.

### 4.3. Vitamin D_2_ or D_3_?

This study identified, for the first time, an inverse relationship between equine serum concentrations of 25OHD_2_ and 25OHD_3_. This has been reported in humans, whereby oral D_2_ or D_3_ supplementation resulted in a linear reduction in serum concentrations of 25OHD_3_ and 25OHD_2_, respectively [27,28,29]. The mechanism underlying this relationship is unclear but may reflect competition between 25OHD_2_ and 25OHD_3_ for interaction with transport binding proteins and metabolising enzymes [27]. It has been proposed that the additional methyl group on carbon 24 of D_2_ influences its affinity for binding proteins and vitamin D receptors, which are critical steps in the activation of the final di-hydroxylated form (1,25(OH)D_2_). Moreover, in the kidneys, an additional hydroxylation of carbon 24 occurs, generating 1,24,25(OH)D_3_. While 1,24,25(OH)D_2_ is no longer active, 1,24,25(OH)D_3_ still maintains biological activity and can bind the vitamin D receptor [30]. These differences are likely to account for the superior potency of D_3_ in some species. It has been proposed that a dynamic balance exists between 25OHD_2_ and 25OHD_3_ metabolites that enables organisms to adapt and maintain adequate vitamin D status despite variability in dietary D_2_ and D_3_ intakes and sun exposure [27]. Given that knowledge of vitamin D metabolism in the horse is limited, and due to the fundamental species-specific differences we have highlighted in this study, further study is warranted to determine whether D_3_ does indeed have superior potency in the horse.

While this study suggests that non-grazing HK racehorses should receive vitamin D supplementation to maintain an adequate vitamin D status for optimal health and athletic performance, further study is required to determine the optimal dose and form of supplementation. In the present study, D_3_ supplementation provided an apparently adequate vitamin D status for most HK racehorses. The HK racehorses included in this study were fed large amounts of concentrate feeds (6–7 kg /horse/d), which provides 6600–14700 IU/d of D_3_. If the average racehorse bodyweight is 500–550 kg, this amount of concentrate alone should provide 12–28 IU/kg BW/d of D_3_. This exceeds the requirements recommended by the National Research Council (NRC) on Nutrients Requirements of Horses [15] (i.e., 6.6 IU/kg for maintenance requirements and 14.7 IU/kg for growing horses of 19–24 months of age), but is well within the upper safety limit of 44 IU/kg/d. It should be noted that the NRC recommendations were extrapolated from an in vivo experimental study conducted in 1979 [31] where circulating vitamin D metabolites were not measured. In light of more recent advances in equine research, up to date and evidence-based guidelines are much needed to better inform daily dietary requirements for vitamin D for sedentary and athletic horses. In humans, oral daily supplementation of 1000–4000 IU/d vitamin D (either D_2_ or D_3_) is sufficient to maintain serum concentrations of 25(OH)D above the threshold for deficiency, which is 75 nmol/L [30]. Interestingly, while on average the horses included in this study received much higher amounts of supplemental D_3_, their total 25(OH)D concentrations did not exceed 29.1 nmol/L. Several factors, such as the molecular form of vitamin D supplementation (i.e., D_2_ vs. D_3_, hydroxylated vs. non-hydroxylated), the food matrix (oil vs. powder), composition of the meal and individual characteristics (e.g., disease state), are likely to influence bioavailability of this nutrient [32]. To the authors’ knowledge, none of these factors has been investigated in the horse. Given that horses do not synthesise relevant amounts of endogenous 25OHD_3_, vitamin D_2_ supplementation may be a more natural approach to efficiently and safely provide supplemental vitamin D to horses, since naturally managed horses rely mostly on 25OHD_2_ derived from dietary forage. Furthermore, D_3_ supplementation potentially has a negative impact on serum 25OHD_2_ concentrations, given the inverse relationship identified herein between the serum concentrations of 25OHD_2_ and 25OHD_3_. While toxicity has been induced in horses using experimental administration of high doses of D_2_ or D_3_ [33,34], there are insufficient data to inform the relative safety of supplemental D_2_ and D_3_ and guide recommendations for supplementation. When the relative toxicity of equimolar high doses (33,000 IU/kg) of D_2_ or D_3_ was compared using only two horses, the D_3_ supplemented horse had a more profound increase in serum concentrations of vitamin D metabolites, and more marked clinical abnormalities and tissue calcification [34]. Further work is required to determine whether supplementation with D_2_, the source of vitamin D in naturally managed horses, is a more appropriate and safer form for horses than D_3_.

### 4.4. The Role of Vitamin D in Equine Musculoskeletal Health and Athletic Performance

The vitamin D status of the racehorses in this study may have been influenced by their high intensity exercise regimens, since experimental high intensity exercise causes a reduction in equine serum 25(OH)D concentrations brought about by unknown mechanisms [10]. Similarly, human elite athletes, especially those exercising for prolonged periods indoors, are at risk of having vitamin D deficiency/insufficiency [35]. The importance of this pleiotropic hormone in skeletal muscle physiology and athletic performance has been widely investigated in other species but not in the horse. Human beings with vitamin D deficiency disorders including rickets and osteomalacia not only suffer skeletal pathologies, but also have skeletal muscle atrophy, myopathies, reduced muscle strength, prolonged time to peak muscle contraction and prolonged time to muscle relaxation [36,37]. Vitamin D is also involved in maintaining adequate calcium availability to support myofiber contraction, and has some local effects via the vitamin D receptors expressed on skeletal muscle cells [10,38,39]. Vitamin D is also thought to stimulate muscle regeneration and to optimise muscle cell metabolism, especially in fast twitch type II fibres, which could be relevant to racing Thoroughbred horses. In some studies of human athletes, vitamin D supplementation increased muscle strength, particularly in the lower limb muscles [40] and in those individuals with the lowest serum concentrations of 25(OH)D [35,41,42,43]. The effect of vitamin D status on racehorse athletic performance clearly warrants investigation. Finally, there is a growing body of evidence demonstrating the beneficial role of vitamin D supplementation on skeletal health and its protective effect against skeletal injuries in human athletes [43]. The incidence of non-catastrophic and catastrophic fractures in HK racehorses is 2.2 per 1000 racing starts and 0.6 per 1000 racing starts, respectively [44]. While vitamin D deficiency and reduced bone density appear to be risk factors for stress fractures in human athletes [45], increased bone density is observed at the most common sites of fracture in equine athletes suggesting that there may be inter-species differences in the mechanisms underlying fractures in human and equine athletes [46,47]. Furthermore, vitamin D appears to have a less important role in regulating calcium metabolism in horses when compared with humans, given that equine intestinal calcium absorption is poorly regulated and urinary calcium excretion is very efficient [21]. The unique nature of vitamin D biology in equids means that caution must be used when making inter-species comparisons regarding the role of vitamin D in bone metabolism and pathology. Investigation of the effect of vitamin D status on bone metabolism and pathology in racehorses is, therefore, clearly warranted.

## 5. Conclusions

Stabled, non-grazing Thoroughbred racehorses should receive vitamin D supplementation to maintain an adequate vitamin D status, because management and occupational factors predispose them to low serum concentrations of 25OHD_2_ and total 25OHD. Further work is required to determine the optimal dose and form of vitamin D supplementation for racehorses. While variable levels of D_3_ supplementation yielded an apparently adequate vitamin D status in most of the Thoroughbred racehorses in this study, the inverse relationship identified between 25OHD_2_ and 25OHD_3_ raises the possibility that oral D_3_ supplementation has a negative impact on serum concentrations of 25OHD_2_. D_2_ supplementation may have advantages because it is the main source of vitamin D in naturally managed horses. Further study is also required to determine the threshold concentrations of serum vitamin D metabolites which indicate deficiency, and whether vitamin D supplementation can improve racehorse health and athletic performance.

## Figures and Tables

**Table 1:** Serum concentrations of 25OHD_2_, 25OHD_3_ and total 25OHD (nmol/L) at the first sampling for 79 HK racehorses and for 22 UK racehorses. ^A^ Significant inter-group difference (*p* < 0.001).

	HK				UK	
nmol/L	25(OH)D_2_	25(OH)D_3_	25(OH)D_tot_	25(OH)D_2_	25(OH)D_3_	25(OH)D_tot_
Median	3.4 ^A^	8.0	12.5 ^A^	7.4 ^A^	8.5	15.8 ^A^
Mean ± SD	4.0 ± 2.97	9.4 ± 5.61	13.4 ± 5.01	8.1 ± 2.20	8.6 ± 3.22	16.7 ± 3.51
Range	<0.5–12.7	1.8–28.6	4.3–29.1	5.6–12.1	3.7–16.3	9.9–26.8
Q_1_–Q_3_	1.6–6.0	5.7–11.5	10.3–15.0	6.1–10.6	6.2–10.2	13.2–19.7

**Table 2:** Serum concentrations of 25OHD_2_, 25OHD_3_ and total 25OHD (nmol/L) for HK racehorses (*n* = 41) at two different time points. ^A^ Significant intragroup difference (*p* < 0.001).

		First Sample	Second Sample
25(OH)D_2_ ^A^	Mean ± SD	5.9 ± 2.6	3.6 ± 2.1
	Median (range)	5.8 (0.5–12.8)	3.5 (0.5–10.1)
	Q1–Q3	4.0–7.4	2.4–4.8
25(OH)D_3_	Mean ± SD	6.4 ± 2.1	7.8 ± 5.2
	Median (range)	6.0 (11.0–1.8)	7.2 (2.1–33.9)
	Q1–Q3	5.1–7.7	4.7–9.4
25(OH)D_tot_	Mean ± SD	12.3 ± 3.5	11.4 ± 4.9
	Median (range)	12.5	10.4 (4.4–34.4)
	Q1–Q3	10.4–14.0	8.5–13.6

**Table 3:** Coefficients calculated for the variables retained in the multivariate linear regression having as outcome total 25(OH)D.

Variables Retained	Regression Coefficient (β)	Standard Error
Ln25(OH)D_3_	0.985	0.024
LnDuration in HK	−0.330	0.056
intercept	1.220	0.107
*p* value	<0.001	
Pearson’s coefficient	0.827	

Variables were transformed into a logarithmic scale.

## Data Availability

All study data are available in Table S1.

## Supplementary Materials

The following supporting information can be downloaded at: https://www.mdpi.com/article/10.3390/ani13132145/s1, Table S1: Full study data set.
